# Can We Exploit β-Lactamases Intrinsic Dynamics for Designing More Effective Inhibitors?

**DOI:** 10.3390/antibiotics9110833

**Published:** 2020-11-21

**Authors:** Eleonora Gianquinto, Donatella Tondi, Giulia D’Arrigo, Loretta Lazzarato, Francesca Spyrakis

**Affiliations:** 1 Department of Drug Science and Technology, University of Turin, via Giuria 9, 10125 Turin, Italy; giulia.darrigo@unito.it (G.D.); loretta.lazzarato@unito.it (L.L.); 2 Department of Life Sciences, University of Modena and Reggio Emilia, Via Campi 103, 41125 Modena, Italy; donatella.tondi@unimore.it

**Keywords:** antimicrobial resistance, β-lactamases, carbapenemases, protein flexibility, intrinsic dynamics, KPC-2, metallo β-lactamases, allosteric enzyme inhibitors, drug design

## Abstract

β-lactamases (BLs) represent the most frequent cause of antimicrobial resistance in Gram-negative bacteria. Despite the continuous efforts in the development of BL inhibitors (BLIs), new BLs able to hydrolyze the last developed antibiotics rapidly emerge. Moreover, the insurgence rate of effective mutations is far higher than the release of BLIs able to counteract them. This results in a shortage of antibiotics that is menacing the effective treating of infectious diseases. The situation is made even worse by the co-expression in bacteria of BLs with different mechanisms and hydrolysis spectra, and by the lack of inhibitors able to hit them all. Differently from other targets, BL flexibility has not been deeply exploited for drug design, possibly because of the small protein size, for their apparent rigidity and their high fold conservation. In this mini-review, we discuss the evidence for BL binding site dynamics being crucial for catalytic efficiency, mutation effect, and for the design of new inhibitors. Then, we report on identified allosteric sites in BLs and on possible allosteric inhibitors, as a strategy to overcome the frequent occurrence of mutations in BLs and the difficulty of competing efficaciously with substrates. Nevertheless, allosteric inhibitors could work synergistically with traditional inhibitors, increasing the chances of restoring bacterial susceptibility towards available antibiotics.

## 1. Introduction

Antimicrobial resistance (AMR) is critically threatening the treatment of a large range of infections caused by bacteria able to inactivate even last resort antibiotics [1]. In the last years, antibiotic-resistance has become quite challenging for the human population and has raised the attention of the World Health Organization [2,3] and of different country governments worldwide [4,5,6].

Among bacteria, resistance is often caused by the overexpression of BLs (β-lactamases) able to hydrolyze and inactivate a vast number of β-lactam antibiotics [7,8]. BLs are classified as serine-BLs (SBLs), using a catalytic serine for antibiotic hydrolysis, and metallo-BLs (MBLs), having one/two zinc ions in the binding site and a hydroxyl ion as active nucleophile [9,10]. SBLs include Ambler classes A, C and D [11,12,13], while MBLs include class B, in turn separated in the three subgroups B1, B2 and B3. BLs can be encoded by both chromosomal and plasmid-mediated genes [13,14].

Extended-spectrum BL (ESBL) [15] and carbapenemases [16,17] are among the most worrisome BLs, being able to hydrolyze, respectively, last generation cephalosporins and a broad variety of β-lactams, including carbapenems, cephalosporins and aztreonam. Carbapenemases have rapidly spread and disseminated worldwide and are compromising the efficacy of carbapenems, the last resort β-lactams used in the clinic for treating the most serious infections. As a matter of fact, carbapenemases are currently among the most relevant/studied targets for the development of new antibacterials. They include, for class A, the chromosomally-encoded and clavulanic acid-inhibited Imipenem-hydrolyzing beta-lactamase (IMI), the Not metalloenzyme carbapenemase of class A (NMC-A), the *Serratia fonticola* resistant to carbapenem BL (SFC-1) and *Serratia marcescens* enzyme (SME), the plasmid-encoded *Klebsiella pneumoniae* carbapenemase (KPC) and Guiana Extended Spectrum (GES)-type enzymes; for class C, only the plasmid-mediated AmpC β-lactamase active on cephamycins (CMY-10), for class D, the acquired OXAcillinase carbapenemase (OXA)-type enzymes. For class B, that includes the most clinically-significant carbapenemases, the New Delhi Metallo-beta-lactamase 1 (NDM-1), the IMiPenemase (IMP) and Verona Integron-encoded Metallo-beta-lactamase (VIM) series have been reported worldwide. Class B enzymes are both plasmid- and integron-located, and are able to inactivate all available β-lactams, with the only exception of aztreonam [18].

Intriguingly, the broad spectrum of action in BL enzymes is often mediated by a single mutation in the catalytic site that can allow for different possible substrate orientations, orchestrated motions of key residues, and subtle changes in the reorganization of waters involved in hydrolysis. As a consequence, a deeper investigation of BL mechanisms of reaction and of their ability to rapidly broaden the range of substrates, as well as the analysis of the enzyme intrinsic dynamics, is fundamental for understanding the rapid onset of resistance and for the design of more effective inhibitors. Moreover, the identification of allosteric sites communicating with the orthosteric one would allow the design of allosteric inhibitors less susceptible to intrinsic and acquired resistance.

## 2. BL Binding Site Flexibility

It is well known that enzymes require a certain level of dynamics to properly work and perform the reactions they catalyze. It has been recently observed that an increased flexibility and hydrophobicity is responsible for the wide spectrum of action of most relevant carbapenemases, irrespectively of the class they belong to [19]. For example, KPC-2 carbapenemase presents a more open and hydrophobic active site with respect to other class A BLs. Similarly, NDM-1 is characterized by a flat pocket lined by many hydrophobic residues [20]. These properties represent an evolutionary advantage for bacteria harboring broad-spectrum BLs, allowing the accommodation of antibiotics, different for size and chemistry. Most importantly, they could be exploited for the design of new effective inhibitors.

### 2.1. Class A Flexibility

**Figure 1:**
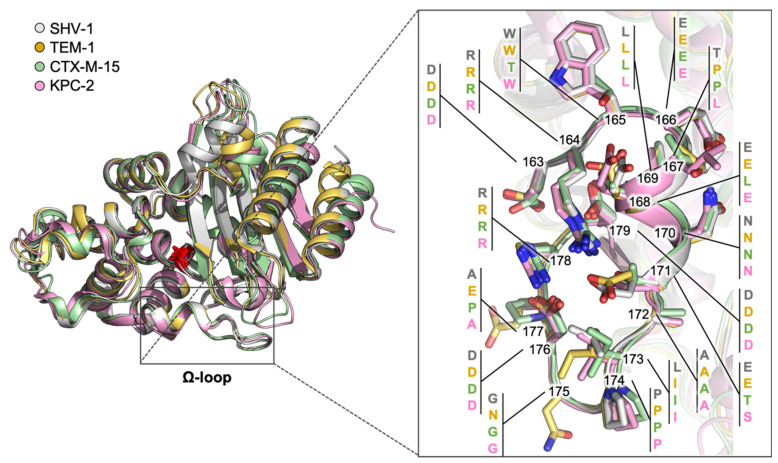
Alignment of class A BLs in cartoon representation: SHV-1 (gray), TEM-1 (dark yellow), CTX-M-15 (green), KPC-2 (pink) (PDB IDs: 4zam, 1ero, 4hbt, 3rxw, respectively). A close-up on the Ω-loop is reported in the right panel: residues are labeled and shown as capped sticks. Each residue number has been linked to four residue names, one for each aligned BL, with a consistent color code. Residues are numbered according to the standard numbering scheme [28].

In class A BLs, the flexibility of the catalytic site is mostly located at the level of the Ω-loop [21]. This spans from residue 164 to 179 and lines part of the active site (Figure 1). It includes Glu166 and Asn170, which participate in acyl-enzyme formation and hydrolysis by means of a water molecule that acts through a proton bypass and that is stably present and well-ordered in the binding site [22]. Glu166 plays its catalytic role by changing location with respect to the other catalytic residues, thanks to the flexibility of the binding site. Interestingly, the Ω-loop is a peculiarity of class A BLs, while it is not present in the original penicillin binding proteins (PBPs). Both loop sequence and conformation are quite conserved in all class A BLs, including Temoneira BL (TEM)-, Sulfhydryl reagent variable (SHV)-, Active on cefotaxime from Munich (CTX-M)- and KPC- types [23]. One of the most frequently mutated residues is Arg164, often substituted with Ser/His/Cys, transforming BLs in ESBLs able to hydrolyze second-to-fourth generation cephalosporins. This is due to the increased volume of the catalytic site, able to host bulky substrates as cephalosporins [24,25]. The substitution of Arg induces the loss of the ionic contact with Asp179, possibly allowing a more rapid water exchange and a consequent larger mobility of the same loop [26]. Considering its role in antibiotic binding and hydrolysis, its high conservation and immunogenicity [27], the Ω-loop could be considered an interesting target for the design of allosteric inhibitors to be used in combination with currently available antibiotics (see paragraph 3.1.1).

KPC-2 is the most worrisome class A BLs, and is responsible for carbapenem resistance in the majority of Gram-negative bacteria [29,30]. The capability of this enzyme to hydrolyze β-lactam antibiotics is strictly connected to the deacylation of the covalent intermediate that is formed during the catalytic process. Apparently, the deacylation occurs when the oxyanion hole, formed by the main nitrogen atoms of Ser70 and Thr237, is properly occupied. Accordingly, the absence of the oxyanion hole should correspond to catalytically non-permissive states of the enzyme [31,32,33]. Cortina et al. deeply investigated the transition/equilibrium between catalytically permissive and non-permissive states of the oxyanion hole, by means of extensive plain and enhanced MD simulations [34]. They first identified key distances for a proper organization of the permissive state, all involving Trp105 and Ser130, which contact the substrate through hydrogen bonds and hydrophobic interactions. Then, they observed that, in non-permissive states, all distances increase, preventing the correct interaction with the oxyanion hole. These non-permissive conformations all present a wider binding cavity in which the loop bearing Trp105 and the SDN loop (residues 130–132) move away from the drug (meropenem in this study), which might lose the proper orientation for being hydrolyzed. The correlation between the loss of catalytic efficiency and the mutation of Trp105 suggested the residue could play a pivotal role in controlling the transition from catalytically permissive and non-permissive states and, consequently, in carbapenem drug resistance.

**Figure 2:**
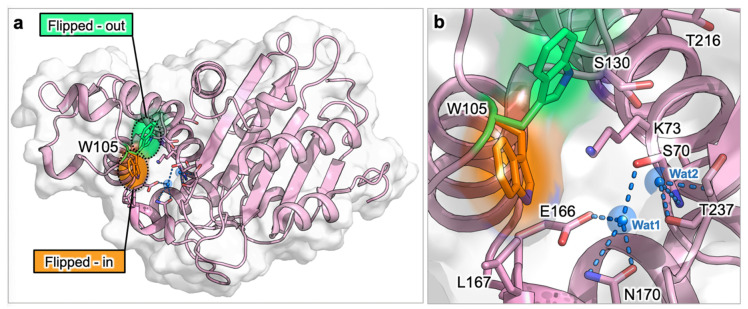
Flipped-in and flipped-out conformations of Trp105 in KPC-2 (PDB ID: 5ul8). (**a**) Overall view of KPC-2. (**b**) Close-up of KPC-2 active site. In the most stable conformation (green), Trp105 side chain is close to Thr216, while in the flipped-in conformation (orange), the indole ring approaches Leu167. Protein is represented in pink cartoon and gray surface, important active site residues are labelled and shown as sticks. Relevant waters are depicted as blue spheres, and labelled: the deacylation water (Wat1) is coordinated by Glu166 and Asn170, while Wat2 occupies the oxyanion hole created by backbone nitrogens of Ser70 and Thr237.

The key role played by Trp105 has been also highlighted by Galdadas et al., who aligned more than 80 class A β-lactamase members and identified two hydrophobic networks: one (the α-network) linking the α helices surrounding the active site, the other (the β-network) connecting the β-sheets with the protein C-terminus. Interestingly, residues in the β-network correspond to the main cryptic site (A1) described in paragraph 3.1.1 [35]. Even if the residues of these hydrophobic networks may vary across different class A β-lactamases, their conserved hydrophobicity is essential for the correct positioning of catalytic residues Ser70-X-X-Lys73. In the same work, the authors performed molecular dynamics simulations and expressed mutants (L102T, I108N and L102T-I108N) of KPC-2, with the aim of disrupting the most important nodes in the hydrophobic α-network. Noteworthy, MIC evaluations showed that mutations increased the susceptibility to penicillins, cephalosphorins, monobactams, and carbapenems, alone or in combination with β-lactamase inhibitors. The authors postulated that mutations inhibited KPC-2 by affecting the position of the catalytic key residue Trp105, which may assume a “flipped-in” or “flipped-out” conformation (Figure 2). In fact, in wild-type KPC-2 the most stable conformation of Trp105 is the “flipped-out” state, which allows a larger binding hinge for accommodating the substrate. On the contrary, mutations in the α-network stabilized a “flipped-in” conformation, with Trp105 exposed to the bulk, leading to the deformation of the two helices shaping the active site (α3 and α4).

Despite the mentioned flexibility, KPC-2 is one of the most stable among class A carbapenemases. Mehta et al. performed stability assays on a series of KPC-2 and nine clinical isolated variants, bearing mutations at key residues 104, 240 and 274 [36,37]. They measured the thermal stability of the enzyme by circular dichroism spectroscopy and found that, even if the mutations decrease the protein stability, mutated KPC still retains higher stability than other class A BLs as TEM-1 or CTX-M-14. Some of the observed mutations allow KPC to expand the activity spectrum also to ceftazidime. Thus, the higher stability of KPC-2 represents a buffer and an evolutionary advantage for the acquisition of multiple destabilizing substitutions that increase its catalytic activity [38]. Indeed, the advantage of a high stability has been often recognized as a favorable condition for the selection of mutants with high catalytic efficiency [39].

The effect of mutations has been deeply investigated for other class A BLs as TEM-1 [40,41,42]. Not all mutations conferring resistance are located close to the active site, but they often occupy regions with medium or high flexibility able to allosterically communicate with it. It seems that nature prefers to change more flexible regions rather than rigid position in catalytic sites, acting in an indirect way on the catalytic efficiency. As substitutions bringing new functions to enzymes are often thermodynamically destabilizing, compensatory stabilizing mutations are needed to maintain folding and function [43].

### 2.2. Class D Flexibility

In response to the selective pressure played by antibiotics, class D BLs adopted a type of evolutionary adaptation similar to class A BLs [44]. Several variants of OXA-23, OXA-24/40 and OXA-51/66 have demonstrated larger substrate specificity, spanning across penicillins, cephalosporins, carbapenems and monobactams [45,46]. Mutations generally occur at the level of the loops lining the active site: the Ω-loop (residues 151–173, [47]), the P loop (residues 93–117, [48]) and the β6β7 loop (residues 220–226, [49]). In the latter, residues Phe110 and Met221, forming a hydrophobic bridge at the top of the binding site, are responsible for increasing the enzyme activity towards carbapenems and for decreasing the binding affinity for bulkier β-lactams, not allowed in such a narrow cavity [45,46]. The OXA-239 variant, isolated from *Acinetobacter baumannii* and evolved from OXA-23 [50], presents three substitutions at the binding site, i.e., S109L, D222N, P225S, and shows higher catalytic efficiency towards cephalosporins and the monobactam aztreonam, while decreased enzyme *k_cat_/K_M_* values towards carbapenems [51]. It was already demonstrated that the substitution of proline at position 225 induces the β6β7 loop to assume a new conformation able to accommodate bulkier substrates [46]. Indeed, the P225S mutation widened the active site cavity and D222N stabilized this new conformation. In the X-ray structures of apo OXA-23 (4k0x) and of OXA-239 in complex with imipenem (5wib), doripenem (5wi7) and cephotaxime (5wi3), the β6β7 loop presents *B*-factors higher than for the rest of the protein, in agreement with the loop higher flexibility. A similar effect was observed for the OXA-160 variant, in which the P227S substitution enlarges the catalytic efficiency to aztreonam and ceftazidime [51]. While the primary mutations (D222N and P225S in OXA-239) in the β6β7 loop increase the binding site flexibility and, consequently the capability of accommodating larger substrates, they also generate a loss in the enzyme stability [52]. Interestingly, secondary mutations partially restore the enzyme stability, while maintaining the effect of the primary ones [53]. Indeed, the S109L substitution demonstrated to have a slight stabilizing effect [51].

### 2.3. Class B (MBL) Flexibility

In this review we only consider MBL B1 subclass, which includes the most widespread carbapenemases. These present, usually, two zinc atoms in the active site, which is located at the interface between two five-stranded antiparallel β-sheets. The active site is delimited by the loops L3 and L10 (residues 57–68 and 223–242, respectively, according to the standard class B BL numbering scheme). Despite their low sequence identity, the overall αβ/βα sandwich fold of MBLs is quite conserved, and there is a common thread throughout the dynamics of these worrisome enzymes.

As we recently reported, the flexibility of NDM-1, which is necessary to perform the hydrolytic reaction and incremented by the high mutation rate, is also responsible for the development of resistant bacteria [54]. The large and shallow active sites of NDM-1 enable the accommodation of almost all families of β-lactam antibiotics; moreover, the reorganization of water molecules and the loop adjustment have made this enzyme very effective and, consequently, the design of inhibitors extremely urgent but challenging. In particular, the flexibility of the Met67-Phe70 region significantly increases the binding site plasticity and allows the accommodation of a number of different β-lactams, with possibly higher affinity [55,56]. Indeed, it is well known that loops L3 and L10 largely change their conformation according to the ligands in the binding site [57], thus playing a fundamental role in substrate binding and stabilization [20,58]. For a deeper description of these aspects in NDM-1, we refer to Linciano et al. [54].

**Figure 3:**
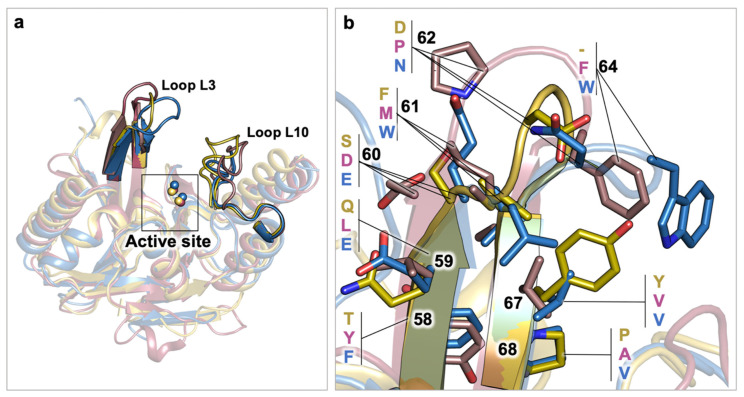
Structural comparison among subclass B1 BLs: VIM-2 (yellow, PDB ID: 4nq2), NDM-1 (pink, PDB ID: 3spu), IMP-1(blue, PDB ID: 1ddk). (**a**) Superposition highlighting loops L3 and L10. Proteins are shown as cartoon, zinc atoms in the active site are represented as spheres. (**b**) Close-up of loop L3. Relevant residues are highlighted in sticks, the residue numbering follows the general class B BL scheme. Residue labels are aligned for each residue identifier.

Clinically-relevant VIM and IMP series, such as VIM-1/2 and IMP-1/2, have been also investigated, searching for structural elements affecting their activity through dynamic relationships. In particular, Yamaguchi et al. highlighted that IMP-1 enzyme is more efficient than IMP-2, possibly because of several factors affecting the flexibility of the active site or the L3 loop (Figure 3) [59]. As a matter of fact, the lower IMP-2 catalytic efficiency could be reasoned by analyzing residues Ser261-Ser262 and the hydrogen bond they establish constraining the active site. Conversely, at the corresponding position in IMP-1, the lack of hydrogen bonds between Pro261-Ser262 guarantees higher degrees of freedom to efficaciously perform the catalysis. The L3 loop is a second critical factor explaining the differences between IMP-1 and IMP-2. The apical portion of L3 loop in IMP-1/2 comprises a Gly63-Trp64-Gly65 (GWG) segment that can adopt an open conformation, widening the binding pocket, or a closed conformation, stabilizing the ligand in the active site. In particular, the side chain of residue Trp64 may be exposed to the solvent when recruiting the ligand and may subsequently rotate to better accommodate it in the active site. In the case of IMP-1, the open conformation has been observed in the apo enzyme, while the closed conformation is prevalent in ternary complexes (PDB ID: 1ddk). In contrast, IMP-2 was crystallized in the closed conformation in absence of ligands (PDB ID: 4ubq). As residues in the active sites are completely conserved, the different behavior of loop L3 between IMP-1 and IMP-2 has been attributed to a proline at position 68, which has been observed as the key residue contributing to maintaining the stability of the L3 loop. Variations at this position have been associated to a higher flexibility of the L3 loop, decreasing the catalytic efficiency, also in VIM enzymes. Indeed, Borra et al. observed that, among other variations, a serine rather than a proline residue at position 68 in VIM-7 contributed to increase the flexibility of loop L3 [60].

In stark contrast, NDM-1 enzyme presents an Ala68 residue, not following the proline conservation at position 68 (Figure 3). To finally clarify the role and functioning of loop L3 in relevant subclass B1 BLs, Vila and coworkers expressed and crystallized the NDM-1 A68P variant, and two engineered NDM-1 with L3 loops from IMP-1 and VIM-2 enzymes [61]. In this work, the open or closed conformation of L3 was demonstrated to depend on the sequence itself, rather than on the protein architecture. Overall, engineered L3 NDM-1 variants showed consistent differences in catalytic mechanism, but no relevant changes in substrate profile with respect to wild-type NDM-1. While loop L3 has been proven a key element in modulating the accumulation of the reaction intermediate, loop L10 has been investigated for its contribution to differential inhibition profiles. In particular, Tyr224 in VIM-2 has been pointed out as a main factor hindering the accommodation of bulky substrates, which are allowed with Leu224 in VIM-5 binding site [62]. Additionally, in VIM-2 residue, Glu225 has been reported to electrostatically interact with Arg228, partially neutralizing its charge and rigidifying the active site. Hence, differently to the mainly hydrophobic L3 loop, in subclass B1, loop L10 can establish polar interactions with other residues, shaping the binding pocket, or with the substrate, stabilizing the binding orientation.

## 3. Allosteric Regulation in BLs: Potential Advantages and Challenges

Allosteric binding events occur when a protein changes its functioning upon ligand binding to a secondary site, distant from the active one. Interestingly, some allosteric sites may be cryptic, as they remain hidden in X-ray apo structures and may open only upon ligand binding. While the clinical benefit of allosteric activators in BLs may not be of relevance, allosteric inhibitors may offer several advantages, the most relevant one being eluding the competition with the substrate. From a drug design point of view, in SBLs, competitive inhibitors are generally conceived for mimicking the tetrahedral transition state, whose charge can be challenging for further ADME optimization. In MBLs, active site inhibitors can act, disrupting the active nucleophile in the active site or, more often, contain zinc coordinating moieties that may exert off-target toxicity in host zinc enzymes and can be limitedly modulated for adjusting the ADME profile [54]. Recent studies demonstrated that common inhibition strategies can be overcome by the design of non-covalent cross-class inhibitors able to affect the catalytic efficiency of both SBLs and MBLs [63,64]. However, the availability of allosteric inhibitors would be highly beneficial because of the potential synergistic effect with more “traditional” inhibitors, or for the restoration of β-lactam antibiotic activity.

### 3.1. Allosteric Effectors in Class A BLs

**Figure 4:**
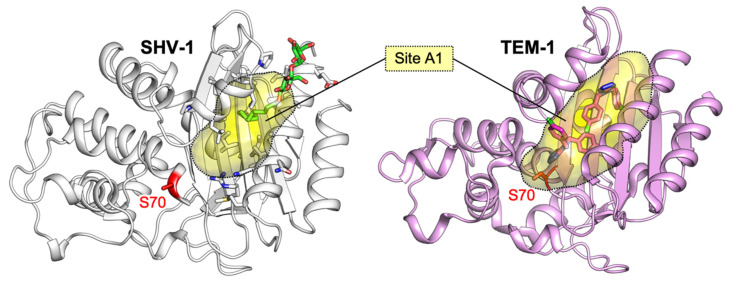
Allosteric site A1 in SHV-1 (gray, PDB ID: 1shv) and TEM-1 (pink, PDB ID: 1pzo) class A BLs.

**Figure 5:**
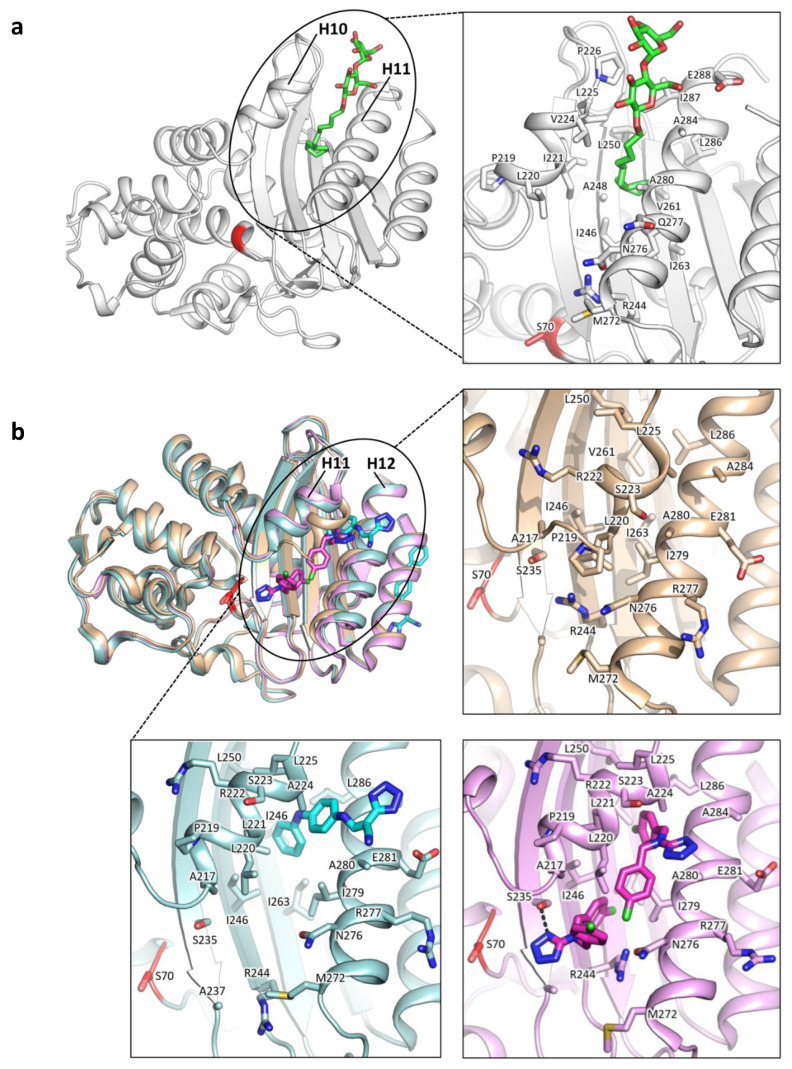
Allosteric effectors in site A1 of BLs SHV-1 and TEM-1. (**a**) SHV-1 X-ray structure (PDB IDs: 1shv) with Cymal-6 (green) bound to the cryptic site A1. (**b**) Superposition among two holo and an apo TEM-1 X-ray structures (PDB IDs: 1pzp (teal), 1pzo (pink), and 1yt4 (light orange), respectively). Proteins are represented in cartoon, while ligands are depicted in bright-colored sticks (Cymal-6, green; FTA, cyan; CBT, magenta). Catalytic Ser70 is highlighted in red as a reference for the position of the active site, while the cryptic site A1 area is approximated by an oval shape. For each X-ray structure in both (**a**,**b**), a close-up of ligand binding modes in the cryptic site is reported, with residues labelled with one letter code and depicted in capped sticks. For clarity, water molecules have been omitted. Residues are numbered according to the standard numbering scheme [28].

Class A allosteric effectors have been investigated since the discovery of a cryptic site in SHV-1 (PDB ID: 1shv; [65]). In his work, Kuzin et al. serendipitously found a detergent molecule 16 Å away from the active site, in a cavity lined by H10, H11 and beta sheets β3, β4, β5. This allosteric site (referred here as site A1, Figure 4) was detected by observing a bound molecule of Cymal-6, whose hydrophobic tail intercalated between helices H10 and H11, while the sugar moiety interacted with polar side chains and the bulk waters (Figure 5a). Interestingly, the binding of Cymal-6 caused a slight reduction in the hydrolytic rate of nitrocefin and penicillin-G (20% and 25%, respectively).

A few years later, the same regulatory site A1 was accidentally identified in TEM-1 by Horn and Shoichet, during the search for BL inhibitors (Figure 4, [42]). In particular, the authors found that two compounds were able to inhibit TEM-1 with a non-competitive and reversible mechanism, which was subsequently characterized by crystallographic studies (PDB IDs: 1pzo, 1pzp; Figure 5b). The two identified inhibitors FTA and CBT opened the site A1 and displayed a K_i_ in the high micromolar range (490 ± 40 µM and 480 ± 20 µM, respectively). This slight inhibitory activity has been linked to the unzipping of H11 (residues 219–226) and H12 (residues 271–289), drifting away Leu220 and Asn276, and consequently loosening Arg244 from the overall structure packing. Arg244 has a crucial role in class A BL catalysis, and its displacement may moderately affect enzymatic activity (Figure 5b). However, it is still unclear whether site A1 may be successfully targeted, even if it offers a large hydrophobic area, potential polar anchors on the surface and a characteristic shape.

A structural analogue of FTA, PA-34 (4-oxo-4-((4-(2,4-dichlorophenoxy)phenyl)amino)butanoic acid) has been reported by Grigorenko et al. to inhibit TEM-171 with a K_i_ = 88 µM [66]. Notwithstanding the structural similarity to the allosteric effector FTA, the authors argued that PA-34 inhibits TEM-171, hampering the entrance to the active site rather than binding to site A1.

Lately, Hart et al. performed a structure-based drug design campaign for identifying TEM-1 allosteric effectors [67]. Starting from a commercial library, the authors carried out a virtual screening using the cryptic site A1 from available X-ray structures and from Markov State Models, yielding two activators and an inhibitor. The authors proved the binding of the three effectors to A1 by site-directed mutagenesis of TEM-1 and docking studies, however no X-ray structure of co-crystals has been reported. Disappointingly, two out of the three tested compounds were racemic mixtures, so that the stereochemistry of binders remained unspecified. Lastly, a work by Avci et al. reported an in silico pipeline of virtual screening and docking for selecting possible binders to class A cryptic site A1 [68]. The structure and score of the ten top-ranked compounds were reported, however, no in vitro validation against class A BLs was carried out.

A comparison of class A BLs co-crystallized with allosteric effectors is reported in Figure 5, while Table 1 sums up relevant information about all allosteric effectors described and characterized against class A BLs.

Allosteric regulation in class A BLs has been further explored by the engineering of TEM-1 BL [69]. In their work, Volkov et al. created, by site-directed mutagenesis BlaKr, an engineered TEM-1 with an allosteric site for aminoglycosides. Insertions and mutations in BlaKr, with respect to the wild-type enzyme, included loops L1, L2, L3 and point mutation E104K, which shaped a serendipitous binding site for kanamycin. Even if co-crystals with kanamycin are not available to date [70], the crystal structure of BlaKr is available in the RCSB (PDB ID: 2v1z, 2v20).

#### 3.1.1. Identification of Cryptic Allosteric Sites in Class A BLs

As cryptic sites are, per definition, not detectable in apo crystal structures, *in silico* simulations have a key role in studying their architecture and behavior in presence or absence of their allosteric effectors. Indeed, many algorithms and approaches have been proposed to identify pockets that, otherwise, might not be visible [71,72,73].

Specifically, Bowman and Geissler used TEM-1 BL as a proof of concept for demonstrating that allosteric sites may be predicted by equilibrium fluctuations of the apo protein [74]. In fact, even if not visible in apo crystal structures, cryptic pockets may be prospectively detected by the sampling of plain extensive molecular dynamics simulations. The authors simulated TEM-1 in absence or presence of the CBT allosteric ligand (Figure 5b, Table 1). The presence of CBT favored and stabilized the opening of the allosteric site A1, reflecting an induced-fit contribution to the binding mechanism. However, the transient opening of the allosteric pocket was observed even in absence of the allosteric effector, suggesting that the conformational selection mechanism may have a leading role. Comparing holo and apo TEM-1 structures, the authors noticed that the binding of the allosteric effector does not induce substantial displacement of the backbone. Hence, the communication between the allosteric and the active site could be mediated by side chain rotations. For supporting this hypothesis, Bowman and Geissler pointed out a community of residues between the active and the allosteric site A1, whose rotameric states were coupled. Moreover, the residues coupling the allosteric site A1 and the active site were found to form part of other minor and smaller transient pockets that, according to the authors, could be targeted by affecting the activity of TEM-1. In a subsequent study, the authors, using an M182T TEM-1 clinically relevant mutant, aimed at identifying allosteric sites other than A1 [75]. Upon mutation to cysteine of Leu286 (a residue in the known cryptic site A1), thiol labelling of Cys286 induced a three-fold reduction in activity. Moreover, using the same methodology, the opening of two additional minor cryptic sites was detected at the level of residues Ala232 and Ala249, and Ser203, respectively. Another study by Bowman and coworkers [76] used exposons (i.e., “clusters of residues that undergo cooperative changes in their solvent exposure”) as a predictive method for finding allosteric sites. This method, first validated on TEM-1 BL, was able to find the known cryptic site A1. In a second moment, exposons were prospectively used on CTX-M-15 and TEM-1 for finding new cryptic sites, and identifying a pocket under the Ω-loop. Specifically, upon thiol labelling of Cys69, a 15-fold decrease in CTX-M-15 catalytic efficiency was recorded. The corresponding pocket in TEM-1 was investigated by mutating Ser243 to Cys and, by thiol labelling, which led to an unexpected 3.75-fold increase in catalytic efficiency. The authors suggested that thiol labelling of this pocket in TEM-1 stabilized a closed conformation, while in CTX-M-15, the same experiments stabilized an open conformation. Consequently, this cryptic site may enhance class A BL catalytic activity when closed and impair it when open. Interestingly, this would be in accordance with the activating and inhibiting effect of allosteric effectors reported by Hart et al. [67].

#### 3.1.2. Relevant Residues Mediating Allosteric Regulation in Class A BLs

**Figure 6:**
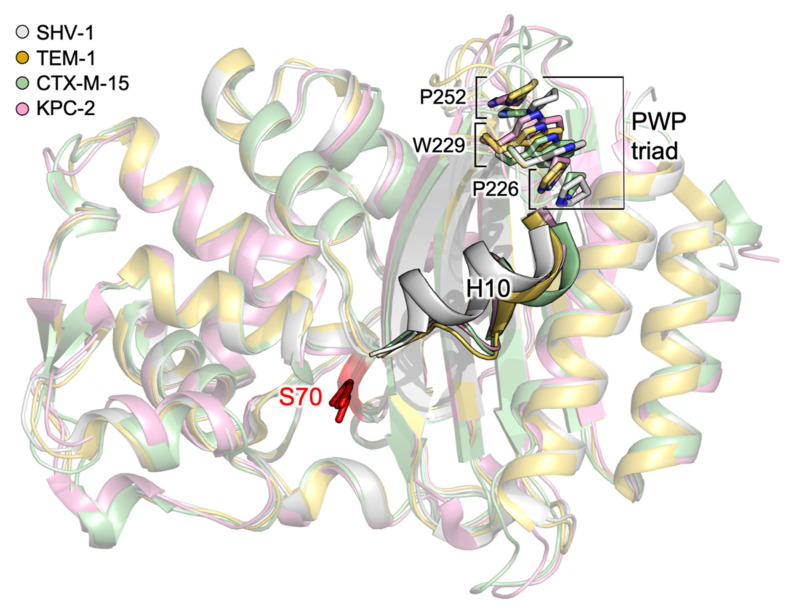
PWP triad in class A BLs. Pro226, Trp229, Pro252 and the catalytic serine are shown in capped sticks. Helix 10 is displayed in cartoon. SHV-1 (gray), TEM-1 (dark yellow), CTX-M-15 (green), KPC-2 (pink) (PDB IDs: 4zam, 1ero, 4hbt, 3rxw, respectively).

Allosteric regulation of class A BLs has been thoroughly explored in the last decade for helping the design of more potent allosteric effectors, and for a better understanding of how mutations can affect enzyme activity. Meneksedag et al. started by observing that the interaction with the BL inhibitor protein (BLIP) causes flexibility loss in H10, which is part of site A1 [77]. In particular, they noted that Trp229 is highly conserved and packed between two conserved proline residues. Molecular dynamics simulation of wild-type SHV-1 and TEM-1, and W229A mutants, were carried out alone or in complex with BLIP. The main outcome reported is that the stacking of Pro226–Trp229–Pro252 (also known as the PWP triad) is essential for H10 stability, and H10 and Trp229 are critical for communication between the known cryptic site A1 and the orthosteric one (Figure 6). Avci et al. analyzed the essential conservation of the PWP triad by creating point mutations of all three residues to Ala, and by further testing the W->Y, W->P substitutions [78]. Interestingly, all mutations led to a drastic decrease in activity, except for W229A, which showed complete loss of activity. Even if mutants did not show secondary structure alterations, their expression level was 10-fold lower with respect to the wild-type. As a consequence, the presence of the PWP triad is mandatory to conserve the activity. Upon sequence alignment, the authors noticed that the PWP at N-terminus of H10 helix triad is unique to class A BLs, however, class C AmpC possesses a similar tryptophane-proline-rich region in a corresponding area.

Lately, Huang et al. published a work demonstrating how increased flexibility of H10 favors the binding of BLIP, and the consequent BL inactivation [79]. This result was obtained by observing an increased H10 mobility in TEM-1, with respect to other two BLs (PC1 and SHV-1), in deuterion exchange experiments.

A work by Latallo et al. presented interesting highlights regarding alternative allosteric pathways in CTX-M-9 [80]. By comparing the sequence of KPC-2 and CTX-M-9, the authors generated 125 virtual point mutants of CTX-M-9, one for each different residue with respect to KPC-2. The mutants were submitted to extensive MD simulations, and subsequently ranked according to their capability of stabilizing the deacylation transition state. Five top-ranked and an arbitrary selection of mid-ranked mutants (specifically L48A, A140K, T165W, T158E, A219H, S220R, N271D, S281A) were expressed in *E. coli*, and their increased resistance to meropenem and cefotaxime was tested. The three mutants showing highest resistance were purified and characterized for understanding the underlying allosteric pathway, as the point mutations were distant to the active site. Of these three mutants, only T165W and L48A were successfully crystallized at high resolution (PDB ID: 5kpu and 5kmt, respectively), but did not show any change in structural features with respect to the wild-type CTX-M-9 (RMSD = 0.3 Å). After the measurement of the root mean square fluctuation (RMSF) of heavy atoms along the MD simulations, the authors suggest that T165W and L48A mutations may affect the drug–enzyme interaction by increasing the conformational flexibility in loop 103–105 and residue N170, both critical in ligand binding and hydrolysis. Lastly, a machine-learning method was applied to MD simulation snapshots, for investigating the active site residues more sensitive to S281A, T165W and L48A mutations. The results for the acyl-enzyme highlighted a pivotal role for residues Asn104, Glu166 and Lys234, whose position was heavily affected by mutations.

Allosteric sites found in class A BLs cannot be repurposed in class B BLs, nor in class D BLs, because of their different fold that lacks the H10 helix.

### 3.2. Allosteric Effectors and Mechanisms in Class B BLs

Allosteric inhibition is the least exploited mechanism to target MBLs, due to two major pitfalls. No allosteric mechanisms have been reported for this class of BLs, and the sequence variability of possible exosites would make the design of small, broad-spectrum molecules nearly impossible. Three azolylthioacetamide derivatives reported by Xiang et al. were characterized as inhibitors with mixed inhibition mechanism (competitive and uncompetitive), however, their uncompetitive inhibition mechanism and binding site has not been reported [81]. At the time of writing, allosteric inhibition has proven a viable strategy only by using macromolecules, such as DNA nanoribbons, DNA aptamers, camelid nanobodies, graphene oxides and nanotubes [82,83,84,85].

### 3.3. Allosteric Effectors and Mechanisms in Class C BLs

Allosteric pathways or effectors in class C and D have not been reported in the literature, however, cooperativity correlation areas (i.e., pairwise mechanical couplings within structure) across four AmpC enzymes have been reported by Brown et al. Specifically, the authors noticed the existence in AmpC of three flexible correlated regions, the first two lining the catalytic Ser64, the third one being the Ω-loop [86].

## 4. Conclusions

As the necessity of new antibiotics is more and more compelling, new design strategies have to be developed. The capability of carbapenemases to hydrolyze nearly all kinds of antibiotics seems to be strictly related to a binding site highly hydrophobic and flexible. Thus, the knowledge of BLs active site dynamics is fundamental to predict the binding of new ligands/inhibitors and to guide their development and optimization. As well, the identification, and prediction, of cryptic sites may open the way for the development of allosteric inhibitors able to affect antibiotic binding, possibly restoring their activity, and to synergize the action of classical inhibitors.

Several *in silico* studies successfully managed to unravel allosteric mechanisms and pockets, proving that computational techniques can have not only a hypothesis-generating, but also a predictive role in this field. Among all the possible and transient allosteric pockets reported by the literature, only one cryptic but druggable site (here named site A1) has been widely recognized in the α-helical domain of class A BLs. Small molecules binding to this site slightly reduced or, in some cases, enhanced enzymatic activity and, thus, were identified as allosteric effectors. However, given the variety of scaffolds reported, several questions remain open. There is no consensus about the chemical features a ligand should have for behaving as allosteric effector, and the rationale for obtaining allosteric inhibitors (rather than activators) is still missing and requires further investigation.

Allosteric inhibitors for class A BLs cannot be repurposed for class B and D members, because of their different fold lacking H10, but there might be a chance for targeting class C BLs, which share some structural and dynamic similarities with class A enzymes. On a separate note, macromolecules such as engineered BLIP, nucleic acids, antibodies or polymers may be an alternative way for studying allosteric inhibition of BLs.

## Figures and Tables

**Table 1:** Summary table reporting name, structure, inhibition/activation profile and reference of class A allosteric effectors. Stereochemistry has been explicitly indicated except for racemic mixtures, whose chiral centers have been marked by (*).

Name	Compound Structure	BL Inhibition (µM)	References	PDB ID
**Cymal-6**	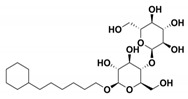	IC_50_ = 100 [68] vs. TEM-1 and 20–25% reaction rate decrease vs. SHV-1 [65]	[65]	1shv
**FTA**	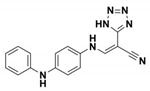	K_i_ = 490 ± 40 vs. TEM-1	[42]	1pzp
**CBT**	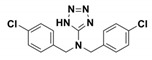	K_i_ = 480 ± 20 vs. TEM-1	[42]	1pzo
**NSC 341597**	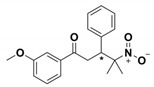	EC_50_ = 57 ± 3 vs. TEM-1	[67]	/
**Name**	**Compound structure**	**BL activation (µM)**	**References**	**PDB ID**
**NSC 333009**	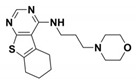	EC_50_ = 63 ± 9 *vs* TEM-1	[67]	/
**NSC 350086**	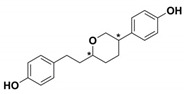	EC_50_ = 162 ± 15 *vs* TEM-1	[67]	/
